# Management of a Modern Operating Theatre

**Published:** 1903-04-25

**Authors:** A. Ferguson MacCallan


### Hospital Clinics and Medical Progress.

### MANAGEMENT OF A MODERN OPERATING THEATRE.

By A. Ferguson MacCallan, M.B., B.C. Camb., F.R.C.S. Eng.

Two of the most important factors in securing the success of surgical operations are, firstly, the performance of the operations in a convenient room fitted with suitable appliances, and secondly the proper management and care of the room and of every single thing which comes in contact with the patient during the operation. These two factors I propose to discuss under the heads of The Room and The Management.

### The Room.

The amount of money available for the provision of an operating theatre varies so widely in different places that the subject can only be considered in its broad outlines. Its position should be such that it is easy of access from other parts of the hospital for the ingress and egress of patients in a recumbent posture; it should not have in its vicinity wards used for septic cases, nor lavatories used by patients. It should be lighted by a large window extending as high as the ceiling and looking towards the north; still more light can be obtained by replacing the ceiling for a couple of feet in the immediate vicinity of the window by a glass roof. The ceiling, walls, and floor should be composed of some non-absorbent material, and I suggest mosaic for the floor and opaline glass for the walls and ceiling. Besides the actual operating-room two or three rooms are required *en suite*, and are important adjuncts. The first in importance is a room to be used by the nurses for the preparation and storage of dressings, which should be large enough to contain a steriliser for dressings if there is not an arrangement for this in another place in the hospital. Of secondary importance are (1) a room for the administration of anæsthetics, which should be well ventilated, and fitted with a cupboard for the safe keeping of the anæsthetics and their accessories; (2) a room where the surgeons can remove their coats, fitted with a writing table and a bookcase for the bound volumes of notes relating to every patient who has left the hospital.

The fittings of the theatre and nurses' room must now be discussed. In the theatre the heating should be by hot water or steam-pipes, which should be so arranged as to be easy of access for cleaning purposes; in the nurses' room there should be instead of, or in addition to, the hot pipes, a fireplace. The question of sinks and wash-places is a most important one, and in the theatre they may be arranged thus: (1) For the surgeons the wash-place should be placed on that side of the room which is opposite the window. It should be of the nature of a large shallow porcelain sink somewhat inclined towards one side, so that water instantly flows away when poured in. The flow of hot and cold water should be operated by a foot lever, and should proceed from the same mouth whither each is led by its proper pipe. (2) On the side of the theatre which is nearest to the nurses' room should be (*a*) A wash basin for nurses; (*b*) a sink for washing trays and instruments; (*c*) a bell-mouthed sink for getting rid of foul lotions, etc. (3) On the side of the theatre opposite the nurses' washing-place is the situation to be occupied by the instrument cabinet, and near it should be a wash-place for the person who looks after the instruments. A shelf to carry the instrument-steriliser should be fastened to the wall by brass supports near the instrument cabinet; the shelf itself may be made of copper, as glass is liable to become cracked by the heat of the steriliser. A glass shelf fastened to the wall by brass supports should be placed above the nurses' washing-place to hold porcelain porringers.

The walls of the theatre should be as free as possible from hot and cold water pipes. Water should be delivered to the wash places by pipes coming straight through the walls.

The artificial lighting should be carried out by means of electric light. For the lighting of the room for general purposes there should be a 32-candle-power lamp in the vicinity of the instrument cabinet and another near the nurses' wash-place. Immediately above the head of the operating-table an 8 candle-power lamp should be placed in the ceiling for the purpose of fixation by the patient during squint operations. The light for the actual operation is best supplied from a movable standard lamp, which carries its light on a jointed arm and can be placed in any position. For operations on the eye there must be some means of darkening the theatre by a blind; this may be placed either inside or outside the window. In the former case a dark cotton blind may be used, but is liable to collect dust; in the latter case a wooden blind of the nature of a shop blind may be made use of, but is liable to get out of order.

Into the details of the operating-table I need not enter, as they may be obtained in all varieties, and at almost any cost, of the instrument-makers. It should be capable of being raised and lowered to suit the requirements of different operators, and should be furnished with a lever for the purpose of altering it from a wheeled table to a stable structure on four legs. The instrument cabinet can be chosen from the makers to suit the requirements of the hospital as to size, etc.; if funds allow, it should be composed of glass and brass only.

Four small glass tables on wheels are needed for use as follows:—(1) For the instruments; (2) for the dressings; (3) for the anæsthetics and their accessories; (4) for lotions for the surgeon's hands, sponges, etc.

In the nurses' room there should be a fairly large table on legs, with a porcelain or glass top on which dressings may be cut. For the keeping of towels and of jars for dressings, sponges, ligatures, etc., there should be a large wooden cupboard with glass doors. Another wooden cupboard will be needed for the storage of bowls, etc. Neither of these cupboards should be fixtures.

(*To be continued.*)

